# Renal replacement therapy in an intensive care unit: guidelines from the SRLF-GFRUP consensus conference

**DOI:** 10.1186/s13613-025-01517-0

**Published:** 2025-07-16

**Authors:** Mercè Jourdain, Ines Gragueb Chatti, Brahim Housni, Pierre Jaquet, Mélissa Jezequel, Oumar Kane, Béatrice La Combe, Mickael Landais, Mehdi Marzouk, Etienne de Montmollin, Guillaume Mortamet, Mai-Anh Nay, Charlotte Salmon-Gandonnière, Sophie Perinel-Ragey, Jérôme Rambaud, Joanna Schmitt, Marie Simon, Julie Starck, Arnaud W. Thille, Pierre-François Dequin

**Affiliations:** 1 INSERM U 1190, Translational Research Diabetes-Lille, Intensive Care Unit, Univ-Lille, CHU Lille, Lille, France; 2 Médecine Intensive Réanimation, Hôpital Nord, Assistance Publique-Hôpitaux de Marseille, Marseille, France; 3 Département d’anesthésie réanimation et médecine d’urgence, CHU Souss Massa, Faculté de Médecine et de Pharmacie, Université Ibn ZOHR, Agadir, Morocco; 4 Service de Médecine Intensive Réanimation, Centre Hospitalier Delafontaine, Saint-Denis, France; 5 Unité de Soins Intensifs de Cardiologie, Centre Hospitalier de Saint-Brieuc, Saint-Brieuc, France; 6 Réanimation Polyvalente, CHU de Fann, Université Cheikh Anta Diop, Dakar, Sénégal; 7 Réanimation Polyvalente, Groupe Hospitalier Bretagne Sud, Lorient, France; 8 Service de Réanimation Médico-Chirurgicale Polyvalente, Centre Hospitalier, Le Mans, France; 9 Médecine Intensive Réanimation, Centre Hospitalier D’Arras, Arras, France; 10 Médecine Intensive Réanimation, INSERM UMR 1137, AP-HP, Hôpital Bichat-Claude Bernard, Paris, France; 11 Pediatric Intensive Care Unit, Univ. Grenoble Alps, Grenoble, France; 12 Intensive Et Réanimation, CHU d’Orléans, Orléans, France; 13 Médecine Intensive Réanimation, INSERM CIC 1415, CHRU de Tours, Tours, France; 14 Médecine Intensive Réanimation, CHU Saint Etienne, INSERM 1059, Saint Priest en Jarez, France; 15 CHU Armand-Trousseau, Médecine-Intensive- Réanimation Pédiatrique, INSERM U955, Sorbonne Université, Paris, France; 16 Réanimation Chirurgicale, Hôpital Edouard Herriot, Hospices Civils de Lyon, Lyon, France; 17 Maladies Infectieuses Et Tropicales, Hospices Civils de Lyon, Lyon, France; 18 CHU Armand-Trousseau, Médecine Intensive Réanimation Pédiatrique, Paris, France; 19 Service de Médecine Intensive Réanimation, CHU de Poitiers, Poitiers, France; 20 Inserm UMR 1100, CIC-P 1415 Et Service de Médecine Intensive Réanimation, CHU de Tours, Université de Tours, Tours, France

**Keywords:** Acute renal failure, Extracorporeal renal replacement therapy, Intermittent hemodialysis, Continuous renal replacement therapy, Consensus conference, Guidelines

## Abstract

**Background:**

Although largely used, the place of extracorporeal renal replacement therapy (RRT) in acute kidney injury (AKI) in intensive care unit (ICU) patients has yet to be clarified. The French Intensive Care Society (Société de Réanimation de Langue Française, SRLF) and the French Pediatric Group of Intensive Care and Emergency (Groupe Francophone de Réanimation et d’Urgence Pédiatrique, GFRUP) organized a consensus conference in November 2024.

**Methods:**

A committee, without any conflict of interest (CoI) on the subject, defined seven generic questions and drew up a list of sub questions according to the population, intervention, comparison and outcomes (PICO) model. An independent work group reviewed literature using predefined keywords. The quality of the data was assessed using the GRADE methodology. Eighteen experts in the field from both societies proposed their own answers in a public session and answered questions from the jury (a panel of 14 critical-care medicine physicians and a nurse) and the public. The jury then met for 48 h to write out and vote on its recommendations.

**Results:**

The panel provided 45 statements addressing seven questions. In patients, adults or children, admitted to the ICU with AKI (1) What are the indications for RRT, when should it be initiated, and within what timeframe? (2) What are the advantages/disadvantages of the different RRT modalities in ICU, and based on what criteria should they be chosen? (3) Which dose of dialysis should be prescribed for ICU patients? (4) How to prescribe, adjust and monitor each RRT technique? (5) Which vascular access technique should be preferred (insertion site, catheter type and length)? (6) How to prevent circuit thrombosis? (7) What are the criteria to consider weaning from RRT and how can it be achieved?

**Conclusions:**

These recommendations should optimize the prescription and use of RRT during AKI in ICUs for both adult and pediatric patients.

**Supplementary Information:**

The online version contains supplementary material available at 10.1186/s13613-025-01517-0.

## Introduction and background

Acute kidney injury (AKI) is a common occurrence in intensive care units (ICUs), affecting 40 to 66% of patients admitted, according to the Kidney Disease Improving Global Outcomes (KDIGO) definitions [1]. AKI is associated with increased morbidity and mortality of critically ill patients [2, 3]. Overall hospital mortality for acute renal failure (ARF) in ICU is reported to be at least 60% [4].

Among critically ill patients presenting with AKI, almost 20% will require extracorporeal renal replacement therapies (RRT) [5]. RRT use in ICU settings may be rising, most likely in response to similar trends in AKI, taken together with an ageing population burdened by high prevalence of multi-morbidity and high illness acuity. Intermittent hemodialysis (IHD) and continuous renal replacement therapy (CRRT) are the two main RRT modalities in adult patients with severe AKI.

Notwithstanding the publication of rigorous clinical practice guidelines focused on RRT for AKI aimed at optimizing the quality and reliability of RRT in ICU settings, practice patterns and outcomes continue to show significant variability.

Numerous international societies and the French Intensive Care Society (Société de Réanimation de Langue Française, SRLF) have proposed guidelines RRT in critical care settings. The French guidelines were most recently updated and published ten years ago. The SRLF wished to review these guidelines in light of the latest international publications by means of a consensus conference format with questions on RRT in ICUs addressed specifically to low-income countries and with a specific focus on sustainable development. In association with the French Group of Pediatric Intensive and Emergency Care (Groupe Francophone de Réanimation et d’Urgence Pédiatrique, GFRUP), the SRLF proposed to draw up specific guidelines for the pediatric population.

The present consensus conference aims to provide evidence-based guidelines for using RRT in AKI in adults and pediatric patients, excluding toxicologic use of RRT. These guidelines are intended for healthcare professionals involved in RRT in critical care, and in ICU settings.

## Methods

The SRLF appointed its Reference and Evaluation Committee to organize a consensus conference in conjunction with the GFRUP on RRT in ICUs.

The members of the committee defined seven generic questions (Table 1), each of them being broken down into several sub-questions developed in the Patient, Intervention, Control and Outcome (PICO) format [6] (additional file).

**Table 1:** Questions submitted to the conference panel

1. What are the indications of RRT, when should it be initiated, and within what timeframe ?
2. What are the advantages/disadvantages of the different RRT modalities in ICU, and on what criteria should they be chosen ?
3. Which dose of dialysis should be prescribed for ICU patients ?
4. How to prescribe, adjust and monitor each RRT technique ?
5. Which vascular access technique should be preferred (insertion site, catheter type and length) ?
6. How to prevent circuit thrombosis?
7. What are the criteria to consider the weaning from RRT and how can this be achieved ?

A bibliographic search was carried out by a group of committee members in PubMed (contributors are listed in the additional file). Keywords were defined based on PICO questions. Grading of Recommendations Assessment, Development and Evaluation (GRADE) tables of published data were drawn up [7]. The quality of evidence was assessed according to the type of study for each of the quoted references and then upgraded or downgraded according to the quality of the study methodology. References were grouped according to each outcome. Overall quality of evidence was then determined for each outcome, based on the quality of evidence of each individual reference, the consistency of results between the different studies, whether the evidence was direct or indirect, and cost analysis.

PICO questions were submitted to experts. Two experts in a given field were appointed for each generic question, one experienced in critically ill adult patients, the other in pediatric intensive care. The experts made their own analysis of the literature, and wrote a text for the panel members debating the assigned question, including the most recent scientific data, as well their opinions and arguments. Additionally, two experts from the REAGIR group (REAGIR, SRLF) on ICU-related sustainable development were tasked with the same work in their fields of expertise.

A panel (Consensus Conference Jury) was constituted of 14 members coordinated by a chairperson. Eleven were physicians working in an adult ICU, including two representatives of medium- or low-income countries. One was a nurse experienced in ICU, especially in RRT. Two were physicians working in a pediatric ICU. None of them had any financial or scientific CoI with the theme of the conference. The role of the panel was to provide a consensus text with the conference’s conclusions and recommendations in the form of a clear answer to each of the questions.

A public meeting was held for the experts, the panel members and a large audience of ICU physicians. The experts presented their analyses for the question they were assigned, and addressed the questions and comments of the panel and the public.

After the public meeting, the panel met privately to draft the text answering the questions. Recommendations were formulated according to GRADE methodology. A high quality of evidence led to a strong recommendation for (GRADE 1 +) or against (1-) a specific intervention. A moderate, low or very low quality of evidence led to an optional recommendation for (probably should, 2 +) or against (probably should not, 2-) a specific intervention. In the absence of evidence, no recommendations were made, except where the panel considered a recommendation in the form of a jury opinion (Table 2).

**Table 2:** Recommendation with GRADE methodology

• Grade 1 = High quality of evidence
Strong recommendation for (1+) or against (1−) a specific intervention
• Grade 2 = Moderate quality of evidence
Low or very low quality of evidence led to an optional recommendation for (probably should, 2 +) or against (probably should not, 2−) a specific intervention
• Absence of evidence
No recommendations, except where the panel considered a recommendation in the form of a jury opinion

Each proposed recommendation was presented and discussed individually. The aim was not necessarily to obtain a convergent opinion of the panel members for all the proposals but rather to uncover points of agreement and disagreement or indecision. Each recommendation was then assessed by each panel member and scored individually from 1 (totally disagree) to 9 (strongly agree). The panel score was determined using a GRADE grid [7]. To validate a recommendation, at least 50% of the panel had to agree (score from 7 to 9) and no more than 20% to disagree (score from 1 to 3), after excluding the two most extreme scores. To achieve a strong recommendation, at least 70% of the participants had to agree. If there was no strong agreement, the recommendations were to be reformulated and scored again in order to achieve a consensus. A strong agreement was necessary for validation of panel opinions. In the final analysis, no recommendation required rewriting and a second round of voting. The final text contains the conclusions and recommendations of the conference.

In the absence of specific pediatric literature, the panel has chosen either to apply the adult recommendation to the pediatric population (when there is no obvious difference between adults and children), or to make no recommendation. When needed, a specifically pediatric recommendation was written. Specific pediatric arguments were detailed only when necessary.

## Section 1: RRT indications and time frame

### Question 1

**What are the indications for RRT, when should it be initiated, and within what timeframe?**

**Recommendation 1.1**

**There are no data available to define specific clinical or biological threshold criteria, given the lack of studies evaluating the impact of RRT on mortality or the number of patients undergoing dialysis at 28 or 90 days. (Panel opinion/Low quality of evidence/Strong agreement).**

**Recommendation 1.1A**

**The panel suggests initiating urgent RRT in cases of hyperkalemia > 6.5 mmol/L, notwithstanding adequate medical treatment. They suggest considering the following factors when deciding to initiate RRT for hyperkalemia: anuria, a very high risk of worsening hyperkalemia, and/or electrocardiographic changes. The adult recommendation also applies to the pediatric population. (Panel opinion/Low quality of evidence/Strong agreement).**

**Recommendation 1.1B**

**The panel suggests not using metabolic acidosis as the sole criterion for initiating RRT. The adult recommendation also applies to the pediatric population. (Panel opinion/Low quality of evidence/Strong agreement).**

**Recommendation 1.1C**

**In adult patients, the panel suggests initiating RRT in a patient with AKI and volume overload pulmonary edema causing moderate to severe hypoxemia that persists despite adequate medical treatment. In the pediatric population, the panel suggests considering RRT in cases of AKI with fluid overload exceeding 10% of body weight, despite restricted intake and adequate diuretic treatment, based on clinical impact and the benefit-risk balance. (Panel opinion/Low quality of evidence/Strong agreement).**

**Recommendation 1.1D**

**The panel suggests considering RRT when, without signs of renal recovery, blood urea nitrogen concentration exceeds 40 mmol/L (> 2.4 g/L). The adult recommendation also applies to the pediatric population. (Panel opinion/Low quality of evidence /Strong agreement).**

**Recommendation 1.1E**

**The panel suggests not using a plasma creatinine threshold value to initiate RRT. The adult recommendation also applies to the pediatric population. (Panel opinion/Low quality of evidence/Strong agreement).**

**Recommendation 1.1F**

**The panel suggests contacting a toxicology expert center based on the specific circumstances of acute poisoning cases. (Panel opinion/Low quality of evidence/Strong agreement).**

**Recommendation 1.1F pediatrics**

**In neonatal or late-onset hyperammonemia, RRT should probably be initiated when ammonia levels exceed 500 µmol/L. The panel suggests initiating RRT when ammonia levels are between 250 and 500 µmol/L in case of very early disease onset (< 48 h of birth), significant signs of encephalopathy, or if ammonia increases despite optimal pharmacological therapy management. (Panel opinion/Low quality of evidence/Strong agreement).**

**Arguments:**

There are no data available in the literature to define specific clinical or biological threshold criteria to indicate RRT in AKI. No precise data are available to establish a defined threshold for management of hyperkalemia. However, a potassium level exceeding 6.5 mmol/L despite optimal treatment represents the highest threshold proposed as a criterion for RRT in multicenter randomized trials [8, 9]. In cases of poor clinical tolerance, RRT may be considered at lower thresholds [10]. Situations with a high risk of rapid worsening (Table 3) warrant particular vigilance and may indicate urgent RRT.

**Table 3:** Situations with a high risk of rapid worsening of hyperkalemia

Sustained cellular lysis: • Acute rhabdomyolysis in the context of extensive traumatic injuries • Acute rhabdomyolysis associated with a genetic deficiency (e.g., LPIN1, CPTII…) • Severe tumor lysis syndrome with a very large tumor burden • Extensive tissue ischemia (e.g., mesenteric infarction)
Heat stroke Malignant hyperthermia
Urinary tract obstruction without the possibility of rapid relief of the blockage

In management, AKI, the cause of acidosis, and other organ failures should be considered in view of tailoring interventions. While the data suggest that delaying RRT initiation until pH reaches 7.15 may not worsen mortality [9–11], each case requires careful risk–benefit assessment. The role of RRT in lactic acidosis is disputed; although extracorporeal lactate clearance is possible, it lags behind endogenous production, thereby limiting the efficacy of RRT efficacy. A randomized trial is underway to evaluate whether administering 4.2% bicarbonate improves 90-day mortality in critically ill patients with AKI and metabolic acidosis. Its results may clarify best practices.

In case of AKI and volume overload pulmonary edema causing moderate to severe hypoxemia, the thresholds proposed by the two randomized trials are SpO2 ≤ 95% on 5 L/min of oxygen or PaO_2_/FiO_2_ ratio below 200 mmHg [10, 12]. They should be interpreted with caution and adjusted to each clinical situation, with special attention paid to the progression of respiratory failure. That said, the proposed thresholds underscore the importance of comprehensive assessment and proactive management for patients with complex organ failure.

While there are no solid data in pediatrics, based on the work by Lintz et al. [13], limitation of fluid overload should be a key-objective.

There is no consensus on blood urea nitrogen concentration thresholds for RRT in ICU patients. However, severe manifestations of uremia (encephalopathy, bleeding, pericarditis) justify its initiation. The AKIKI trial used a 40 mmol/L threshold to start RRT in the group randomized to delayed strategy, with no difference in 60-day mortality compared to the group with early strategy [14]. In AKIKI2, the initiation of RRT later, with a threshold set at 50 mmol/L (mean blood urea nitrogen concentration of 43 ± 13 mmol/L) was associated with increased 60-day mortality [14]. Though not the sole trigger, these findings suggest avoiding levels above 40 mmol/L.

Available literature provides no conclusive evidence to establish a specific creatinine threshold that would justify RRT initiation. None of the randomized trials conducted to date have included a specific plasma creatinine level as a criterion.

The considerable diversity of acute poisoning scenarios makes it challenging to adopt a uniform approach for RRT indications in this situation. An international group of experts was formed to establish consensus indications for the use of RRT in cases of acute poisoning. This group, named Extracorporeal Treatments in Poisoning (EXTRIP) (https://www.extrip-workgroup.org), provides recommendations based on thoroughgoing analysis of the literature and collective expertise [15, 16].

Hereditary metabolic diseases are most commonly diagnosed in young infants, and can be associated with very poor outcome [17, 18]. The neurological prognosis is directly correlated to plasma ammonia concentration and its rapid normalization (if ammonia > 1000 µmol/L, a palliative approach may be considered) [19, 20]. Therefore, the response to emergency medical management should rapidly be evaluated and RRT considered in case of insufficiently decreased or increased ammonia levels. Peritoneal dialysis (PD) should only be used if no other form of dialysis is available [18]. The suggested thresholds of 250 and 500 µmol/L are based on case series and expert opinions [21, 22].

**Recommendation 1.2**

**RRT should not be initiated before 72 h in adults with AKI classified as KDIGO 3 in the absence of emergency criteria (e.g., hyperkalemia, severe acidosis, or volume overload pulmonary edema). (Grade 1-/High quality of evidence/Strong agreement). This recommendation also applies to the pediatric population.**

**Arguments:**

This recommendation is supported by large randomized trials. A wait-and-see approach can avoid RRT in 38% to 49% of patients [9, 10, 12]. Mortality at day 28 or 90 does not increase when dialysis is started after 72 h of oliguria [9, 10, 12]. This strategy offers more RRT-free days at day 28 [9, 10], though not at day 90 [12]. It also confers economic and environmental advantages by reducing resource use and environmental impact, without compromising clinical outcomes. In conclusion, in case of non-recovery of renal function or anuria for 72 h, RRT should be considered [15].

## Section 2: Different RRT modalities and modalities of prescription

### Question 2

**What are the advantages/disadvantages of the different RRT modalities in ICU, and based on what criteria should they be chosen?**

**Recommendations 2.1 and 2.2A**

**The different types of RRT (including continuous, intermittent, or hybrid modalities) should probably be considered equivalent in terms of prognostic criteria. (Grade 2 + /Moderate quality of evidence/Strong agreement).**

**Arguments:**

Randomized studies and two meta-analyses (although with heterogeneous definitions of hemodynamic instability) found no significant differences in hemodynamic failure between CRRT, IHD, or hybrid methods [23–28].

The panel suggests considering clearance levels rather than opposing continuous techniques to intermittent or hybrid dialysis. The required intensity of clearance varies depending on the patient's clinical progression. Only one early randomized controlled trial reported increased mortality with CRRT [29], but it did not specify the proportion of patients in shock, and there were numerous crossovers between purification techniques. The following studies, mainly involving patients on vasopressors, showed similar mortality rates at day 28 and day 90 [25, 30–37]. Recent post hoc studies showed divergent mortality results [14, 38, 39]. Results concerning sustained low efficiency dialysis (SLED) are likewise divergent [36, 37]. Two recent meta-analyses confirmed no overall superiority of any technique (continuous or intermittent) [28, 40]. No difference was found between any of the techniques in terms of number of days alive without RRT or renal recovery [30, 36].

**Recommendations 2.1 and 2.2B**

**The panel suggests that the choice of technique should be guided by the expertise of the medical team, the availability of equipment, and its environmental impact. (Panel opinion/Low quality of evidence/Strong agreement).**

**Arguments**:

Regarding cost, which is difficult to estimate precisely, some studies have nevertheless shown that continuous techniques are associated with higher expenses [41, 42]. Environmental impact, which encompasses water use, consumable waste, and anticoagulation choices, also requires careful consideration.

**Recommendations 2.1 and 2.2C pediatrics**

**In pediatrics, the panel recommends using CRRT rather than IHD in the hemodynamically unstable patient. In hemodynamically stable patients, the panel makes no recommendation for one technique rather than another and suggests that the choice of RRT method should be based on the availability of equipment and the technical expertise of the team. (Panel opinion/Low/Strong agreement).**

**Arguments**:

It is technically impossible to perform IHD on children weighing less than six kilograms, and difficult to perform on children less than 10 kg. There are no comparative pediatric studies between the different techniques. However, the use of intermittent or hybrid techniques carries a high risk of hemodynamic disorders due to children’s physiological particularities. The extra-body volume used in intermittent or hybrid techniques is greater than 10% of the child's total blood volume. Data on hybrid techniques in pediatrics are scarce. The study by Sethi et al. [43] included 68 patients from 2010 to 2016, and analysed 211 SLED sessions, including 148 (70.1%) patients receiving inotropic drugs. One study emphasizes the feasibility of SLED in low-income countries [44].

**Recommendation 2.3A**

**Peritoneal Dialysis is probably not recommended for adult patients with AKI when vascular access is available. (Grade 2-/Moderate quality of evidence/Strong Agreement).**

**Recommendation 2.3B**

**The panel suggests that in low and middle-income countries or resource-constrained settings, Peritoneal Dialysis should be considered for adult patients with AKI only if no technique with vascular access is available. (Panel opinion/Low quality of evidence/Strong agreement).**

**Recommendation 2.3 pediatrics**

**In pediatrics, the panel suggests considering PD as an alternative to a vascular approach in the absence of life-threatening ionic disorders, hyperammonemia or digestive lesions. (Panel opinion/Low quality of evidence/Strong Agreement).**

**In pediatrics, the panel suggests considering PD as an alternative to a vascular approach in the absence of life-threatening ionic disorders, hyperammonemia or digestive lesions. (Panel opinion/Low quality of evidence/Strong Agreement).**

**Arguments**

Peritoneal dialysis (PD) has been particularly developed in resource-limited countries and during healthcare crises [45], with specific guidelines [46]. Studies comparing PD with vascular access techniques (continuous or intermittent) report divergent results on clearance efficiency and mortality [18, 28, 47–53]. Some studies excluded emergency epuration criteria due to low clearance of PD [47, 49, 50]. PD appears to be better tolerated hemodynamically [47] but should not replace RRT in emergency cases unless no technique with vascular access is available. During the COVID-19 crisis, PD was used in some ICUs in high-income countries, thereby renewing interest [54–59]. Infectious complications appear similar, though low quality evidence [47–50].

While PD is less expensive, industrially produced solutions are costly and may be unaffordable in resource-limited settings. Locally prepared solutions present quality risks and should only be used when no alternatives are available.

There is no randomized study comparing PD with dialysis using a vascular approach in pediatric ICUs. Pediatric literature consists only in descriptive registry studies (European Dialysis Registry for Acute Renal Failure). PD still has a significant role in pediatrics, as it is used in 30 to 42% of the pediatric patients. PD is an alternative to CRRT with a vascular approach in cases of hemodynamic instability [47, 48, 60], contraindication to anticoagulation, difficulty implementing a central venous line or difficulty accessing RRT with a vascular approach. However, PD is less efficient in achieving rapid correction of life-threatening ionic disorders or congenital hyperammonemia, and cannot be used in cases of digestive ischemia or perforation [49, 50, 60, 61].

### Question 3

**Which dose of dialysis should be prescribed for ICU patients?**

**Recommendation 3.1**

**A maximum effluent dose of 25 ml/kg/h during continuous renal replacement therapy, obtained through filtration and/or diffusion, should be prescribed in adults. (GRADE 1 + /High quality of evidence/Strong agreement).**

**Arguments**

The VA/NIH [62] and RENAL [8] studies did not demonstrate reduced 28-day or 90-day mortality, or a reduced proportion of patients requiring dialysis at 60-day or 90-day using a high dose of prescribed dialysis (> 20–25 ml/kg/h).

In clinical practice, the dose delivered is lower than the prescribed dose, 22 mL/kg/h versus 25 mL/kg/h in RENAL [8]; 19 mL/kg/h versus 20 mL/kg/h in VAH/NIH [66,]. Data comparing lower dialysis doses of less than 20–25 ml/kg/h remain limited and retrospective [63]. Results show that patients treated with low-intensity CRRT (14.3 ml/kg/h) did not exhibit worse outcomes in terms of in-hospital mortality compared to those treated with currently standard intensity (20.4 ml/kg/h). These findings require confirmation in a RCT taking into account sustainable development.

**Recommendation 3.1A pediatrics**

**In pediatrics, the panel suggests prescribing an effluent flow between 25 and 35 ml/kg/h. (Panel opinion/Low quality of evidence/Strong agreement).**

**Recommendation 3.1B pediatrics**

**In pediatric patients suffering from endogenous intoxication with hyperammonemia, the panel suggests prescribing high doses of effluent flow (> 50 ml/kg/h). (Panel opinion/Low quality of evidence/Strong agreement).**

**Recommendation 3.1C pediatrics**

**In pediatric patients with septic shock, the panel suggests prescribing high doses of effluent flow (> 50 ml/kg/h). (Panel opinion/Low quality of evidence/Strong agreement).**

**Arguments**

The effluent dose required in the pediatric population is higher than in adults. This difference is related to a higher circulating plasma mass/weight ratio, e.g. 70–80 ml/kg in infants versus 50 ml/kg in adults. This physiological difference becomes less significant as the child grows, with no age or weight cut-off. The closer the child gets to an adult size, the more we should consider applying the recommendation for adults.

In congenital hyperammonemia, a case series showed improved ammonium and organic acid clearance with effluent flow rates higher than 35 ml/kg/h [21, 64]. According to some case series, these flow rates could even be increased to 3–4 times the standard effluent flow rate [65]. The impact of the rate of ammonia reduction remains a matter of debate. In a multicenter study, Pica et al. showed no difference in outcome and survival, regardless of the type of RRT used [66]. These findings are increasingly challenged by recent multicenter studies showing an improved prognosis in children managed with vascular-access RRT rather than PD [67, 68].

As regards septic shock in pediatric patients, there exist three randomized controlled trials [69–71] and a recent meta-analysis [72] comparing standard and high effluent flow. Cui et al. conducted a single-center study comparing standard dialysate flow (35 ml/kg/h) with high flow (50 to 70 ml/kg/h) in 72 patients in septic shock. They confirmed the feasibility of this technique, as well as a non-significant trend toward reduced mortality [69]. The study by Ning et al. of 47 patients admitted for sepsis randomized them into two groups so as to compare a high effluent flow (> 60 ml/kg/h) with a standard effluent flow (30 ml/kg/h) and showed no difference in survival between the two groups. The authors report significant improvement in the oxygenation index and lactate levels at 72 h of treatment in the high-flow group [70]. As for Meng et al., they randomized 76 children admitted for sepsis into two groups so as to compare a high effluent flow (> 50—100 ml/kg/h) with a standard effluent flow (35—50 ml/kg). Survival was longer in the high effluent flow group (26.3% versus 7.0%, p: 0.033). No other clinical endpoints were reported in this study [71].

**Recommendation 3.2**

**Daily intermittent hemodialysis aimed at increasing the weekly dialysis dose should probably not be performed. (GRADE 2 + /Moderate quality of evidence/Strong agreement). In the absence of data, no pediatric recommendation can be made.**

**Arguments**

The VA/NIH study compared two dose intensities of RRT [62]. The intensive RRT group included patients treated with IHD or SLED on a daily basis (six sessions per week). Conversely, the less intensive dialysis group received three sessions per week. No significant benefit in terms of 60-day mortality or renal recovery was observed in the intensive RRT group. In post-hoc analysis of the subgroup of patients treated exclusively with IHD, there was a significant increase in the number of dialysis-free days and renal recovery at 28 days in the less intensive RRT group [73]. Daily IHD is more expensive and less environmentally friendly than IHD performed three times a week, because the cost of consumables and labor increases in proportion to the number of sessions.

**Recommendation 3.3**

**The panel makes no recommendation concerning rhythm of RRT based on clinical and biological arguments and/or a fixed rhythm of every other day. This applies to the pediatric population. (Insufficient quality of evidence/Strong agreement).**

**Arguments**

There are no studies comparing a systematic IHD schedule with a schedule based on emergency dialysis criteria (fluid overload, acidosis, hyperkalemia, urea). This represents a potential area for research aimed at identifying a minimal effective IHD schedule.

**Recommendation 3.4**

**The panel suggests not exceeding a dialysis dose corresponding to weekly Kt/V of 3.9. (Panel opinion/Low quality of evidence/Strong agreement). As pediatric data are lacking, the panel makes no recommendation.**

**Arguments**

Kt/V (urea) is a normalized value of urea clearance achieved during a session of IHD and developed for chronic hemodialysis. In ICU, the value of Kt/V use has not been demonstrated Kt/V (urea) of 1.3 was the target for IHD in the VA/NIH study [62]. A post-hoc analysis of this study examined the subgroup of patients treated with IHD [73], comparing treatment with IHD three times a week (weekly Kt/V urea target of 3.9), to treatment with IHD six times a week (Kt/V urea target of 7.8). It was not shown that exceeding a Kt/V target of > 3.9 improved renal recovery at Day 28 or according to the number of days without dialysis.

### Question 4

**How to prescribe, adjust and monitor each RRT technique?**

**Recommendation 4.1**

**The panel makes no recommendation concerning the choice between portable osmosis device and online ultrapure water for IHD in adults or pediatrics. (Panel opinion/Insufficient quality of evidence/Strong agreement).**

**Arguments**

There is no study comparing water purification devices in ICUs. The use of online ultrapure water in ICUs is possible with a contamination rate (bacteria, endotoxins) < 1% [74]. Regardless of the device, a water quality monitoring procedure must be implemented. The choice between techniques must consider local factors, particularly the proximity of a chronic dialysis unit with an online ultrapure water treatment device. The costs of the two options, especially in low- and middle-income countries, must be considered.

**Recommendation 4.2A**

**High dialysate sodium concentration should probably be used at initiation of dialysis session to improve hemodynamic tolerance (GRADE 2 + /Moderate quality of evidence/Strong agreement). In pediatrics, the panel makes no recommendation (Insufficient quality of evidence).**

**Recommendation 4.2B**

**A cooler dialysate temperature (-1.5 °C to -2 °C below patient’s temperature) should probably be used to improve hemodynamic tolerance (GRADE 2 + /Moderate quality of evidence/Strong agreement). In pediatrics, the panel makes no recommendation (Insufficient quality of evidence).**

**Recommendation 4.2C**

**The panel makes no recommendation regarding reduced blood flow with extended dialysis session to improve hemodynamic tolerance (Insufficient quality of evidence). In pediatrics, the panel makes no recommendation (Insufficient quality of evidence).**

**Arguments**

Most studies regarding dialysis prescriptions assess intervention bundles, making individual parameter analysis challenging. Two studies reported that initiating dialysis with a high sodium concentration (150–160 mmol/L) and reducing it to 138–140 mmol/L significantly lowered hypotension requiring intervention [75, 76]. Fixed high dialysate sodium concentration (≥ 145 mmol/L) can also improve hemodynamic tolerance [77]. In hyponatremic patients, dialysate sodium concentrations should be adjusted.

A dialysate temperature of 35–35.5 °C compared to 37 °C reduces hypotension while maintaining ultrafiltration targets and without affecting endof-session patient temperature [76, 78]. Caution is needed for awake patients, who may have a low tolerance for colder dialysate.

There is insufficient evidence to determine the hemodynamic effects of extending dialysis from four to six hours with reduced blood flow during IHD.

Data are insufficient to make recommendations for low- and middle-income countries or sustainability. The impact of dialysis settings on mortality and renal recovery remains unstudied.

**Recommendation 4.3**

**The panel suggests not using devices with online monitoring and automatic biofeedback control for intradialytic blood volume and temperature. (Panel opinion/Low quality of evidence/ Strong agreement). For the pediatric population, the panel makes no recommendation. (Insufficient quality of evidence).**

**Recommendation 4.4**

**The panel suggests choosing a low clearance method in patients at risk of intracranial hypertension or with very high blood urea nitrogen to avoid dialysis disequilibrium syndrome. (Panel opinion/Low quality of evidence/Strong agreement). As regards the pediatric population, the panel makes no recommendation. (Insufficient quality of evidence).**

**Recommendation 4.5**

**The panel suggests systematically assessing blood volume and not initiating ultrafiltration if clinical evaluation indicates intravascular hypovolemia. The panel suggests stopping ultrafiltration if hypovolemia symptoms arise during RRT. The adult recommendations also apply to the pediatric population. (Panel opinion/Low quality of evidence/Strong agreement).**

**Arguments**

In ICUs, a single-center, randomized, controlled trial evaluated devices that automatically adjust blood volume and/or temperature, and found that they did not reduce episodes of hypotension or arrhythmias or the number of therapeutic interventions [79]. Until additional data are available, their use does not seem justified.

In AKI patients, rapid osmotic fluctuations, particularly in blood urea nitrogen levels, can increase cerebral edema and intracranial pressure [80]. In high-risk patients, i.e. patients with acute cerebral injury, fulminant hepatitis or very high blood urea nitrogen, we recommend low-dose treatment (CRRT or low-dose dialysis).

The following factors seem associated with the occurrence of hypotension during RRT: epinephrine infusion, low blood pressure, and a positive passive leg raising test. Hypotension is associated with increased 90-day mortality [81].

In pediatrics, CRRT-related hypotension is common [82] and may lead to momentary interruptions of fluid removal during RRT [83]. Risk factors for CRRT-related hypotension include previous hemodynamic instability, circuit priming volume, and blood flow rate [83–86].

**Recommendation 4.6**

**The panel suggests not using high cut-off membranes or other specific RRT membranes. (Panel opinion/Low quality of evidence/Strong agreement). The adult recommendations apply to pediatric population. The panel suggests adapting membranes to the child’s weight. (Panel opinion/Low quality of evidence/Strong agreement).**

**Recommendation 4.7**

**The panel suggests that continuous veno-venous hemodiafiltration (CVVHDF) improves filter life span compared to continuous veno-venous hemofiltration (CVVHF). (Panel opinion/Low quality of evidence/Strong agreement). As relevant data are lacking, the panel makes no recommendation regarding the efficacy of CVVHDF for purifying small molecules, compared to CVVHF. (Panel opinion/Low quality of evidence/Strong agreement). In pediatrics, the panel makes no recommendation (Insufficient quality of evidence).**

**Arguments**

High cut-off membranes and specialized membranes (e.g., heparin-coated or adsorptive) have not proven superior to standard membranes (polysulfone, polymethyl methacrylate). Their use is not justified in terms of sustainability and cost. Additionally, membrane type may affect drug adsorption of drugs, particularly antimicrobials, warranting therapeutic drug monitoring.

In pediatrics, several filter sizes are commercialized for children, including infants (such as the Carpediem hemofilter, Medtronic, Inc., Dublin, Ireland, with a priming volume of 26 to 41 ml). The average duration of small surface area membranes is significantly lower [87].

Two studies comparing CVVHDF and CVVHF found shorter filter life with CVVHF [88, 89]. However, with widespread use of citrate for regional anticoagulation, the difference may become less significant. In addition, HDF is more time-consuming for caregivers, making it challenging in low-income settings. Regarding sustainability, CVVHDF over CVVHF based on filter lifespan alone seems unreasonable.

In pediatrics, a single, low-powered study compared the elimination of small and medium-sized particles according to technique and found comparable elimination on polyacrylonitrile filters used for CVVHF and CVVHDF at 35 ml/kg/hr. The study also found a longer filter operating time for CVVHDF.

## Section 3 Technical aspects

### Question 5

**Which vascular access technique should be preferred (insertion site, catheter type and length) ?**

**Recommendation 5.1A**

**Jugular and femoral venous sites should probably be considered equivalent in terms of infectious complications. (GRADE2 + /Moderate quality of evidence/Strong agreement).**

**Recommendation 5.1B**

**The panel makes no recommendation on choice of insertion site for a dialysis catheter to lower the risk of thrombosis. (Insufficient quality of evidence/Strong agreement).**

**Recommendation 5.1C**

**In order of preference, 1) the right internal jugular site 2) the femoral site 3) the left internal jugular site should probably be preferred to lower the risk of dysfunction. (GRADE2 + /Moderate quality of evidence/Strong agreement).**

**Recommendation 5.1D**

**The subclavian route should probably be avoided for the insertion of dialysis catheters unless there is no alternative. This recommendation also applies to the pediatric population. (GRADE2-/Moderate quality of evidence/Strong agreement).**

**Arguments**

Femoral and jugular sites are the main insertion sites for temporary dialysis catheters. No significant difference in infection risk has been observed between them [90–92], although colonization is lower with a femoral site for BMI < 24 kg/m^2^ and a jugular site for BMI > 28 kg/m^2^ [90].

The risk of venous thrombosis remains low (0.5%) and similar between the two sites [90].

Catheter dysfunction is less frequent with the right jugular site compared to femoral and left jugular sites [93]. A 12F catheter, with length adapted to the insertion site (≥ 24 cm for femoral, 12 cm for right jugular, and 20 cm for left jugular), optimizes blood flow and reduces the risk of dysfunction [94–96]. The femoral site is not recommended in cases of abdominal compartment syndrome. If early rehabilitation is considered, femoral site should be avoided. Finally, spacing between central venous catheter and dialysis catheter should be ensured to prevent drug interference [97].

The subclavian site appears to have a lower infection risk [98] but should be preserved if there is a risk of progression to end-stage renal failure [99, 100], as it entails a risk of subclavian vein stenosis [92].

**Recommendation 5.1E**

**In pediatrics, the panel suggests using the right jugular vein access in children under three years old. In children over three years old, the right jugular vein and a femoral vein access are equivalent. Whatever the patient’s age, the panel suggests respecting a ration size of the catheter/vein diameter < 0.33 in choice of catheter size. (Panel opinion/Low quality of evidence/Strong agreement).**

**Arguments**

In children under three years old, the size of the femoral vein is significantly smaller than that of the jugular veins [101]. Placement of venous access in an internal jugular vein may be associated with improved circuit survival [102, 103].

It is important to note that the ratio of diameter of the catheter to the weight of the patient or size of the vein could be associated with thrombosis occurrence [104, 105]. According to this study, it appears that a catheter-to-vein diameter ratio greater than 0.33 is associated with a higher risk of thrombosis.

**Recommendation 5.2A**

**The panel makes no recommendation on the choice of a 4% citrate or heparin lock (≤ 5000 IU/mL) versus a saline lock so as to lower the risk of dysfunction, infection and thrombosis. This recommendation applies to the pediatric population. (Panel opinion/Insufficient quality of evidence/Strong agreement).**

**Recommendation 5.2B**

**A high concentration of heparin (> 5000 IU/mL) or citrate (> 4%) should probably not be used to achieve a lock. This recommendation also applies to the pediatric population. (GRADE2-/Moderate quality of evidence/Strong agreement).**

**Recommendation 5.2C**

**Heparin locks should probably not be used in patients at risk of hemorrhage. This recommendation also applies to the pediatric population. (GRADE2-/Moderate quality of evidence/Strong agreement).**

**Arguments**

Data on the effectiveness of citrate locks in preventing catheter dysfunction remain limited, with conflicting results. While one study reported a significant reduction in dysfunction with a 47% citrate lock compared to saline, frequency of lock renewal differed from current practices [106]. Other studies confirmed reduced dysfunction with citrate compared to saline or heparin [107], but comparisons between citrate and heparin have yielded mixed results.

Regarding infection, some studies have suggested reduced catheter colonization with citrate without a significant impact on bacteremia [107], while others have found no difference between citrate, heparin, and saline [106, 108]. Additionally, no significant difference in thrombosis rates was observed between 4% citrate and 5000 IU/mL heparin [108].

Heparin doses ≤ 5000 IU/mL are associated with lower bleeding risk, whereas highly concentrated citrate (46.7%) carries a risk of cardiac complications [109]. Several studies have highlighted a reduced bleeding risk with citrate [110, 111], while heparin lock may increase anti-Xa activity and bleeding risk [112].

Use of citrate or heparin should be evaluated from both an economic and an ecological perspective, particularly in resource-limited settings where citrate availability may be restricted.

**Recommendation 5.3A**

**Ethanol locks should probably not be used to lower the risk of infection. This recommendation also applies to the pediatric population. (GRADE2-/Moderate quality of evidence/Strong agreement).**

**Recommendation 5.3B**

**The panel makes no recommendation on use of an antibiotic lock to lower the risk of infection. This recommendation also applies to the pediatric population. (Panel Opinion/Insufficient quality of evidence/Strong agreement).**

**Arguments**

A randomized study found no difference in infectious risk between a 60% ethanol lock and saline solution for dialysis catheters [113]. No data are available on the use of antibiotic locks in ICU patients.

In pediatrics, there are no specific data on dialysis catheters. However, by analogy with other types of catheters, a benefit in using citrate, tauroline and ethanol may reside in the prevention of catheter-related infection [114, 115].

## Section 4: Anticoagulation

### Question 6

**How to prevent circuit thrombosis?**

**Recommendation 6.1**

**In patients at low risk of bleeding, CRRT with regional citrate anticoagulation should probably be preferred over anticoagulation with unfractionated heparin or low molecular weight heparin. This recommendation also applies to the pediatric population. (GRADE 2 + /Moderate quality of evidence/Strong agreement).**

**Arguments**

Meta-analyses of randomized trials in high- and middle-income countries comparing regional citrate anticoagulation with unfractionated or low-molecular-weight heparin or a heparin-protamine combination showed that citrate prolongs circuit lifespan and reduces bleeding risk, with comparable mortality rates [116].

Adverse effects of regional citrate anticoagulation include metabolic alkalosis, hypocalcemia and hypercalcemia, requiring active monitoring. When systemic anticoagulation with heparin is otherwise indicated, the superiority of regional citrate anticoagulation remains unproven.

Regarding sustainability, a study reported a CO₂ footprint of 670 kg for a three-day heparin treatment compared to 3.85 kg for citrate [117].

In pediatrics, no randomized trials have taken place, but observational studies show similar results [118–126]. Only one retrospective pediatric study reports lower mortality with citrate than with unfractionated heparin [127].

**Recommendation 6.2**

**In patients at high risk of bleeding, in the absence of contraindication to citrate, CRRT with regional citrate anticoagulation should probably be preferred to no anticoagulation. (GRADE 2 + /Moderate quality of evidence/Strong agreement). The panel makes no recommendation regarding the pediatric population. (Insufficient quality of evidence/Strong agreement).**

**Arguments**

There exists no consensus on the definition of a high bleeding risk. A meta-analysis found that regional citrate anticoagulation prolongs filter lifespan compared to no anticoagulation [128]. A prospective study reported fewer hemorrhagic events with regional citrate anticoagulation than with no anticoagulation [129], but mortality data are lacking. When systemic anticoagulation with heparin is otherwise indicated, the superiority of regional citrate anticoagulation remains unproven.

Patients with liver cirrhosis or acute liver failure are at increased risk of citrate overdose, requiring adapted protocols targeting a total calcium to ionized calcium ratio below 2.5 [130].

In pediatrics, only one descriptive study has focused on children at hemorrhagic risk, and it suggests that there were no serious complications or significant metabolic disturbances with citrate [131].

**Recommendation 6.3**

**The panel makes no recommendation regarding the choice of systemic anticoagulation with unfractionated or low molecular weight heparin rather than regional citrate anticoagulation in patients with an indication for IHD and a high risk of bleeding. The panel makes no recommendation regarding the pediatric population. (Insufficient quality of evidence/Strong agreement).**

**Arguments**

No robust studies on ICU patients have addressed this question. Additionally, due to a lack of system automation, implementation of regional citrate anticoagulation during IHD is challenging.

## Section 5: RRT weaning

### Question 7

**What are the criteria to consider weaning from RRT and how can it be achieved?**

**Recommendation 7.1**

**The panel suggests assessing the resumption of diuresis to consider weaning from IHD in AKI. (Panel opinion/Low quality of evidence/Strong agreement). The panel makes no recommendation regarding the pediatric population.**

**Arguments**

To date, no study has demonstrated a strong criterion for weaning from RRT. The absence of a clear definition of weaning from RRT prevents homogeneous studies from being conducted [132, 133].

The panel emphasizes the importance of evaluating the kinetics of resumption of diuresis when considering weaning from IHD. Indeed, a retrospective study of 304 patients with AKI requiring RRT in post-operative ICUs reported that oliguria (< 100 ml/8 h; OR 4.17) measured the day after stopping hemodialysis was significantly associated with weaning failure [132].

No blood or urine biomarker has demonstrated a superior predictive value in predicting weaning from IHD. However, measuring the concentration of urinary urea could be of interest in a strategy of weaning from IHD. In a single-center retrospective cohort, daily urinary urea excretion, corresponding to [(urinary urea (mmol/L) x diuresis (L/24 h)) / Patient weight (kg)], was significantly associated with weaning from IHD [134]. The interest of a combination of urinary urea and diuresis in a strategy of weaning from IHD remains to be confirmed.

Unconventional biomarkers such as NGAL, L-FABP, or Cystatin C have not been sufficiently studied during IHD, and they are expensive. They have no place in a strategy of weaning from IHD.

**Recommendation 7.2**

**The panel suggests assessment of diuresis resumption when considering weaning from CRRT. This recommendation also applies to the pediatric population. (GRADE 2 + /Moderate quality of evidence/Strong agreement).**

**Arguments**

Diuresis at the time of stopping CRRT is the first predictive marker of withdrawal to have been described in two studies including more than 500 patients (respective thresholds of 436 ml/day [135] and 191 ml/day [136]; however, the period of successful withdrawal from CRRT was three days, which can be considered too short). More recently, an association between the volume of diuresis in the hours following CRRT cessation and successful weaning from RRT has been highlighted [137, 138]. In pediatrics, only one study [139] has investigated predictive factors for successful weaning from CRRT. Urinary output greater than 0.8 ml/kg/h in the six hours preceding CRRT cessation was predictive of success. Use of diuretics diminished the strength of this association.

At the end of CRRT filter lifespan, the panel suggests not restarting the therapy immediately, in the absence of emergency criteria for RRT, the objective being to enable a possible resumption of diuresis. The few available data suggest that RRT delays recovery of renal function. Gaudry et al. demonstrated that a delayed RRT strategy in ICU patients facilitated earlier recovery of renal function [10].

Unconventional biomarkers such as NGAL, L-FABP, or Cystatin C have not demonstrated any benefit in observational studies [140, 141] and have not been studied in pediatrics.

**Recommendation 7.3**

**The panel suggests daily assessment of the possibility of weaning from CRRT. If available, IHD can be used as a relay. This recommendation also applies to the pediatric population. (Panel opinion/Low quality of evidence/Strong agreement).**

**Arguments**

No prospective randomized study has compared continuation of CRRT until complete weaning with early interruption of CRRT with an IHD relay.

A retrospective trial did not describe increased mortality or hemodynamic instability when switching from CRRT to sustained SLED [142].

Weaning from RRT as early as possible seems desirable. Several studies have reported possibly reduced resumption of diuresis under RRT, especially when intensive hemodialysis is used [10, 143]. Finally, despite a lack of data, the individual impact of CRRT, which requires immobilization of the patient, who may necessitate rehabilitation, the environmental impact of CRRT [144] and its cost [145], call for consideration of weaning from CRRT as early as possible.

In pediatrics, patient weight has an impact on technical possibilities. Below six kg, the use of IHD remains difficult. Prolonged use of IHD has been described in children in centers without access to CRRT but has not been studied as part of a weaning strategy.

## Supplementary Information

Additional file 1: PICO questions.

## Data Availability

Not applicable.

## Abbreviations

AKI: Acute kidney injury
ARF: Acute renal failure
BMI: Body mass index
CoI: Conflict of interest
CRRT: Continuous renal replacement therapy
CVVH: Continuous veno-venous hemofiltration
CVVHD: Continuous veno-venous hemodialysis
CVVHDF: Continuous veno-venous hemodiafiltration
GFRUP: Groupe francophone de réanimation et d’urgence pédiatrique
GRADE: Grading of recommendations assessment development and evaluation
HDF: Hemodiafiltration
ICU: Intensive care unit
IHD: Intermittent hemodialysis
KDIGO: Kidney disease improving global outcomes
MV: Mechanical ventilation
PICO: Patient intervention control outcome
PD: Peritoneal dialysis
RCA: Regional citrate anticoagulation
RCT: Randomized controlled trial
REAGIR: Réanimation globale innovante réduisant la production de gaz à effet de serre
RRT: Renal replacement therapy
SLED: Sustained low efficiency dialysis
SRLF: Société́ de réanimation de langue française
