# Supplementary material for: Renal replacement therapy in an intensive care unit: guidelines from the SRLF-GFRUP consensus conference
Source: Ann Intensive Care. 2025 Jul 16;15:100. doi: 10.1186/s13613-025-01517-0 (PMC12267776; doi:10.1186/s13613-025-01517-0)
Supplement: Supplementary file 1 — Additional file 1: PICO questions. [file 13613_2025_1517_MOESM1_ESM.docx]

**PICO questions**

**Q1. What are the indications for RRT, when should it be initiated, and within what timeframe?**

**1.1: indication**

**P**: In ICU patients, with or without AKI

**I**: Did application of threshold-based clinico-biological criteria (e.g., hourly urine output, potassium levels, pH, PaO2/FiO2, creatinine or blood urea nitrogen levels, plasma concentrations of dialyzable toxins or endogenous dialyzable metabolites such as ammonium) help to determine the indication for RRT

**C**: Compared to an empirical decision not based on threshold criteria (at the discretion of clinicians)

**O**: Impact 28-day mortality? 90-day mortality? The proportion of patients requiring dialysis at 90 days? The number of dialysis-free days measured at 28 days and 90 days?

**1.2 Delay**

P: In ICU patients with AKI KDIGO 3 but without criteria for urgent dialysis (hyperkalemia, deep acidosis, pulmonary pressure edema)

I. Did postponed dialysis until criteria for urgent dialysis appeared

C. Compared to an immediate dialysis strategy (within 12 hours)

O. Impact 28-day mortality? 90-day mortality? The proportion of patients requiring dialysis at 90 days? The number of dialysis-free days measured at 28 days and 90 days?

**Q2. What are the advantages/disadvantages of the different RRT modalities in ICU, and based on what criteria should they be chosen?**

**2.1 AKI without associated shock**

**P:** In ICU patients with criteria for initiating RRT in AKI without associated shock

**I:** Did CRRT

**C:** Compared to IHD or hybrid dialysis

**O:** Improved hemodynamic tolerance during dialysis sessions? Mortality at Day 28? Mortality at Day 90? Proportion of patients requiring dialysis at Day 90? Number of dialysis-free days at Day 28 and Day 90? Sustainability? Feasibility in low-income countries?

**2.2 AKI with associated shock**

**P:** In ICU patients with criteria for initiating RRT in AKI with associated shock

**I:** Did CRRT

**C:** Compared to IHD or hybrid dialysis

**O:** Improve hemodynamic tolerance during dialysis sessions? Mortality at Day 28? Mortality at Day 90? Proportion of patients requiring dialysis at Day 90? Number of dialysis-free days at Day 28 and Day 90? Sustainability? Feasibility in low-income countries?

**2.3 Peritoneal dialysis (especially in pediatrics and low-income countries):**

**P:** In ICU patients with criteria for initiating RRT in AKI

**I:** Did peritoneal dialysis

**C:** Compared to RRT with vascular access

**O:** Was effective in terms of clearance? to manage hypervolemia? to reduce vascular complications, mechanical complications, and infectious complications? Improved per-dialytic hemodynamic tolerance? Impacted mortality at Day 28 and Day 90? What was the proportion of patients requiring dialysis at Day 90? What was the number of dialysis-free days at Day 28 and Day 90? Was it sustainable? Was it feasible in low-income countries?

**Q3. What dose of dialysis should be prescribed for ICU patients?**

**3.1 Effluent flow rate?**

**P:** In ICU patients with CRRT indication

**I:** Did an effluent flow rate > 25 ml/kg/day (adjusted for or independent of downtime and adjusted weight)

**C:** Compared to an effluent flow rate of 20-25 ml/kg/day (adjusted for or independent of downtime and adjusted weight)

**O:** Improved 28-day mortality? 90-day mortality? The proportion of patients requiring dialysis at 90 days? The number of dialysis-free days measured at 28 days? At 90 days? Sustainability? Feasibility in low-income countries?

**3.2 Frequency?**

**P:** In ICU patients with IHD indication

**I:** Did daily IHD

**C:** Compared to IHD every other day

**O:** Improved 28-day mortality? 90-day mortality? The proportion of patients requiring dialysis at 90 days? The number of dialysis-free days measured at 28 days? At 90 days? Sustainability? Feasibility in low-income countries?

**3.3 On demand**

**P:** In ICU patients with IHD indication

**I:** Did an IHD schedule based on fluid overload, acidosis, hyperkalemia, and blood urea nitrogen levels

**C:** Compared to a systematic schedule of IHD every other day

**O:** Improved 28-day mortality? 90-day mortality? The proportion of patients requiring dialysis at 90 days? The number of dialysis-free days measured at 28 days? At 90 days? Sustainability? Feasibility in low-income countries?

**3.4 Kt/V**

**P:** In ICU patients with AKI receiving IHD

**I:** Did a Kt/V target of >3.9 per week

**C:** Compared to a systematic schedule of IHD 3 times a week (3 sessions at Kt/V 1.3 per week)

**O:** Improved 28-day mortality? 90-day mortality? The proportion of patients requiring dialysis at 90 days? The number of dialysis-free days measured at 28 days? At 90 days? Sustainability? Feasibility in low-income countries?

**Q4. How to prescribe, adjust and monitor each RRT technique?**

**4.1 Water purification device in IHD**

P. In ICU patients with IHD indication

I. Did a portable osmosis device

C. Compared to online ultrapure water

O. Was associated with greater water contamination (bacteria or endotoxins)? More ICU acquired infections? More dialysis complications? Better sustainability? Better feasibility in medium or low-income countries?

**4.2 IHD, hemodynamic tolerance**

P. In ICU patients with IHD indication

I. Did high conductance (sodium concentration of the dialysis fluid) at the initiation of a dialysis session and/or a low dialysate temperature (-1.5 to -2 °C of the patient’s temperature) and/or dialysis duration of at least 6 hours in combination with a blood flow of approximately 150 mL/min

C. Compared to standard conductance (corresponding to the patient's natremia) and a dialysate temperature identical to the patient’s and a dialysis duration of 4 hours in combination with a blood flow of approximately 200-250 mL/min

O. Reduced episodes of perdialytic hypotension? The dose of perdialytic vasopressors? Mortality at 28 days? At 90 days? The proportion of patients requiring dialysis at 90 days? The number of days without dialysis at 28 days? At 90 days? Does it improve sustainability? Is it feasible in low-income countries?

**4.3 IHD, hemodynamic tolerance, automation of settings**

P. In ICU patients with IHD indication

I. Did the automatic adjustment of settings based on continuous monitoring of the patient's blood volume and temperature (feedback)

C. Compared to non-automated settings

O. Reduced episodes of perdialytic hypotension? The dose of perdialytic vasopressors? The mortality rate at 28 days? At 90 days? The proportion of dialysis patients at 90 days? The number of days without dialysis measured at 28 days? At 90 days? Does it improve sustainability? Is it feasible in low-income countries?

**4.4 IHD, hemodynamic tolerance, special cases**

P. In ICU patients for whom IHD is indicated

I. Did the use of specific settings during the first session, especially with very high plasma urea and/or in patients with brain damage

C. Compared to standard settings

O. Improved the hemodynamic and neurological tolerance of this first dialysis session?

**4.5 Hemodynamic tolerance of ultrafiltration**

P. In ICU patients treated with RRT

I. Did prescription of ultrafiltration based on predictive criteria for poor hemodynamic tolerance or on occurrence of perdialytic hypotension

C. Compared to empirical prescription (at clinicians' discretion)

O. Reduced episodes of perdialytic hypotension? The dose of perdialytic vasopressors? Mortality at 28 days? At 90 days? The proportion of patients requiring dialysis at 90 days? The number of days without dialysis measured at28 days? At 90 days?

**4.6 Filters, membranes**

P. In ICU patients with IHD indication

I. Did the choice of a hyperpermeable membrane (or with a very high cut-off) or another specific membrane

C. Compared to a conventional membrane

O. Reduced episodes of perdialytic hypotension? The dose of perdialytic vasopressors? The mortality rate at 28 days? At 90 days? The proportion of patients requiring dialysis at 90 days? The number of days without dialysis measured at 28 days? At 90 days? Does it improve durability? Is it feasible in low-income countries?

**4.7 CHD, HDF vs. HF**

P. In ICU patients with CRRT indication

I. Did the choice of hemodiafiltration (HDF)

C. Compared to hemofiltration (HF)

O. Increased the clearance of small molecules (potassium, creatinine, urea)? The lifespan of haemofilters?

**Q5. Which vascular approach should be preferred in terms of insertion site, type and length of the catheter?**

**5.1 Femoral site**

P. In ICU patients with RRT indication

I. Did insertion of a dialysis catheter in femoral venous site

C. Compared to the internal jugular venous site

O. Influenced catheter contamination and infections? Venous thrombosis at implantation site? Catheter dysfunction?

**5.2 Lock and thrombosis**

P. In ICU patients with a catheter for RRT indication

I. Did use of a citrate "lock"

C. Compared to a "lock" with heparin or saline solution

O. Influenced venous thrombosis at the implantation site? Catheter dysfunctions?

**5.3 Lock and infection/contamination**

P. In ICU patients with a catheter for RRT indication

I. Did use of an antibiotic or antiseptic "lock"

C. Compared to a "lock" with heparin or saline solution

O. Influenced catheter-related bloodstream infections? Catheter contamination?

**Q6. How to prevent circuit thrombosis?**

**6.1 CRRT, low risk of bleeding**

P. In patients at low risk of bleeding with CRRT

I. Did regional anticoagulation with citrate

C. Compared to systemic anticoagulation with unfractionated heparin or low molecular weight heparin

O. Had an impact on circuit lifespan? Hemorrhagic complications? Metabolic complications (metabolic alkalosis, hypocalcaemia, hypomagnesemia)? Durability? Feasibility in low-income countries?

**6.2 CRRT, risk of bleeding**

P. In patients at risk of bleeding on CRRT

I. Did regional citrate anticoagulation (to be adapted to the patient’s condition, particularly in cases of hepatocellular insufficiency)

C. Compared to no anticoagulation

O. Had an impact on circuit lifespan? Hemorrhagic complications? Metabolic complications (metabolic alkalosis, hypocalcaemia, hypomagnesemia)? Mortality at 28 days? At 90 days)?

**6.3 Intermittent RRT**

P. In patients at risk of bleeding during IHD

I. Did systemic anticoagulation with unfractionated heparin or low molecular weight heparin

C. Compared to no anticoagulation or regional anticoagulation

O. Had an impact on treatment efficiency? Circuit lifespan? Hemorrhagic complications? Metabolic complications (metabolic alkalosis, hypocalcemia, hypomagnesemia)? Durability? Feasibility in low-income countries?

**Q7. What are the criteria to consider weaning from RRT and how can it be achieved?**

**7.1 When and based on what criteria should weaning from IHD be considered?**

P: In ICU patients with AKI treated with IHD (either the only technique used by the unit or following CRRT)

I: Did weaning from IHD based on spontaneous resumption of diuresis or under loop diuretic therapy, above a given threshold value

C: Compared with weaning from IHD based on blood and/or urine biomarkers (including serum creatinine and measurement or estimation of glomerular filtration rate)

O: Affected dialysis-free days measured at 28 days? 90 days? Proportion of patients on renal replacement therapy at 90 days? MAKE 30? MAKE 90? Mortality at 28 days? 90 days? Sustainability? Feasibility in low-income countries?

**7.2 When and based on what criteria should weaning from CRRT be considered?**

P: In ICU patients with AKI treated with CRRT

I: Did weaning from CRRT based on spontaneous resumption of diuresis or under loop diuretic therapy, above a given threshold value

C: Compared with weaning from CRRT based on blood and/or urine biomarkers (including serum creatinine and measurement or estimation of glomerular filtration rate)

O: Affected dialysis-free days measured at 28 days? 90 days? Proportion of patients on renal replacement therapy at 90 days? MAKE 30? MAKE 90? Mortality at 28 days? 90 days? Sustainability? Feasibility in low-income countries?

**7.3 Relay between IHD and CRRT**

P: In ICU patients with AKI treated with CRRT (if both techniques are available)

I: Did a relay with IHD in the ICU

C: Compared to maintaining CRRT until complete cessation of RRT (or until transfer to nephrology unit)

O: Affected dialysis-free days measured at 28 days? 90 days? Proportion of patients on RRT at 90 days? MAKE 30? MAKE 90? Mortality at 28 days? 90 days? Sustainability? Feasibility in low-income countries?
